# Supplementary material for: Preclinical Evaluation of a Novel Small Molecule Inhibitor of LIM Kinases (LIMK) CEL_Amide in Philadelphia-Chromosome Positive (BCR::ABL+) Acute Lymphoblastic Leukemia (ALL)
Source: J Clin Med. 2022 Nov 15;11(22):6761. doi: 10.3390/jcm11226761 (PMC9692768; doi:10.3390/jcm11226761)
Supplement: Supplementary file 1 [file jcm-11-06761-s001.zip › jcm-2005002-supplementary.pdf]

Table S1 : PCR primers used in this study

Target gene	Sequence
LIMK1 Fw	5'-AAGAATGTGGTGGTGGCTGA-3'
LIMK1 Rv	5'-TTCTTGCGGTCTGGCTTCTT-3'
LIMK2 Fw	5'-TCCCCGAACACTGGACTTTG-3'
LIMK2 Rv	5'-CTCCAGTCTGCAGCAGATGG-3'
GAPDH Fw	5'-GATCCCTCCAAAATCAAGTGG-3'
GAPDH Rv	5'-GGAGGCATTGCTGATGATCT-3'

Table S2: Characteristics of patients

**Patient No.**	**Age**	**Gender**	**WBC G/L**	**BM Blasts%**	**Karyotype**	**WHO 2016**
1	23	F	64	85	6,XX,+8,t(9;22)(q34;q11),-16[12]/47,idem,+10[10]	B-ALL (t9;22)
2	78	F	6	99	Failure	B-ALL (t9;22)
3	54	M	126	83	46,XY,t(9;22)(q34;q11) [21]	B-ALL (t9;22)
4	22	M	22	95	45,XY,-7,t(9;22)(q34;q11)[12]/46,XY[12]	B-ALL (t9;22)

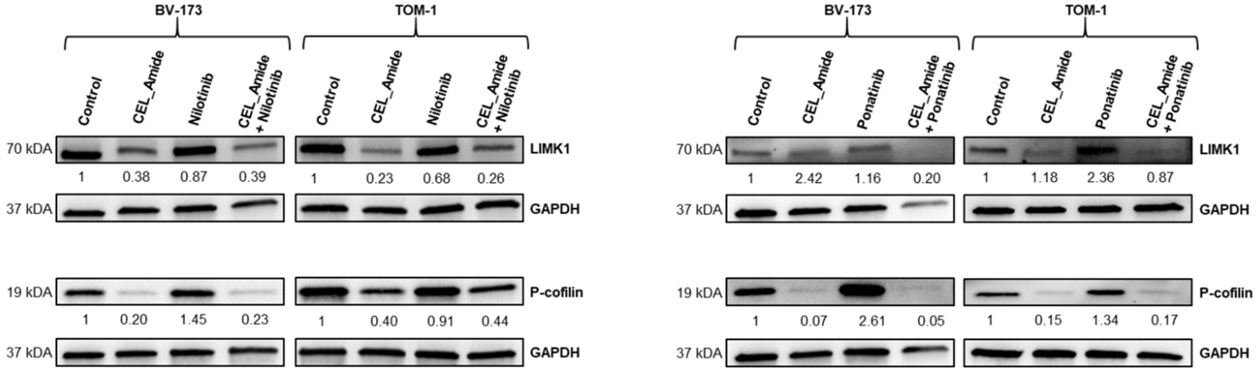

(A) **(B)**

Figure S1: LIMK1, LIMK2, cofilin and phospho-cofilin protein expression in *BCR::ABL*+ B-ALL cell lines after TKI exposure. Western blot showing protein changes of LIMK1 and phospho-cofilin in BV-173 and TOM-1 cell lines after 72h exposure to 1500nM of CEL_Amide or 25nM nilotinib alone or in combination. GAPDH was used as a loading control. One representative experiment out of three is shown (**A**). Western blot showing protein changes of LIMK1 and phospho-cofilin in BV-173 and TOM-1 cell lines after 72h exposure to 1500nM of CEL_Amide or 1nM ponatinib alone or in combination. GAPDH was used as a loading control. One representative experiment out of three is shown (**B**).

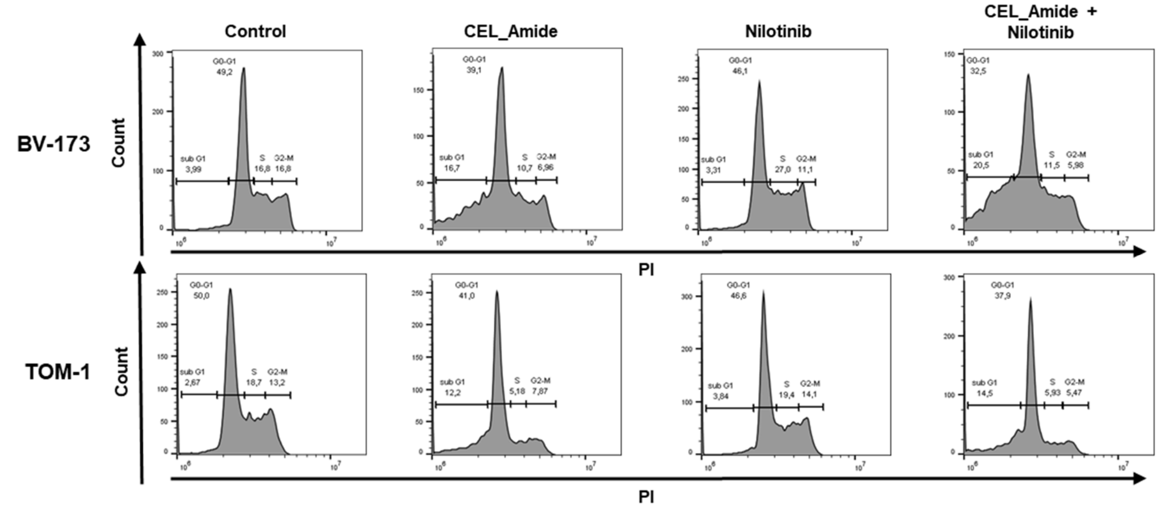

(A)

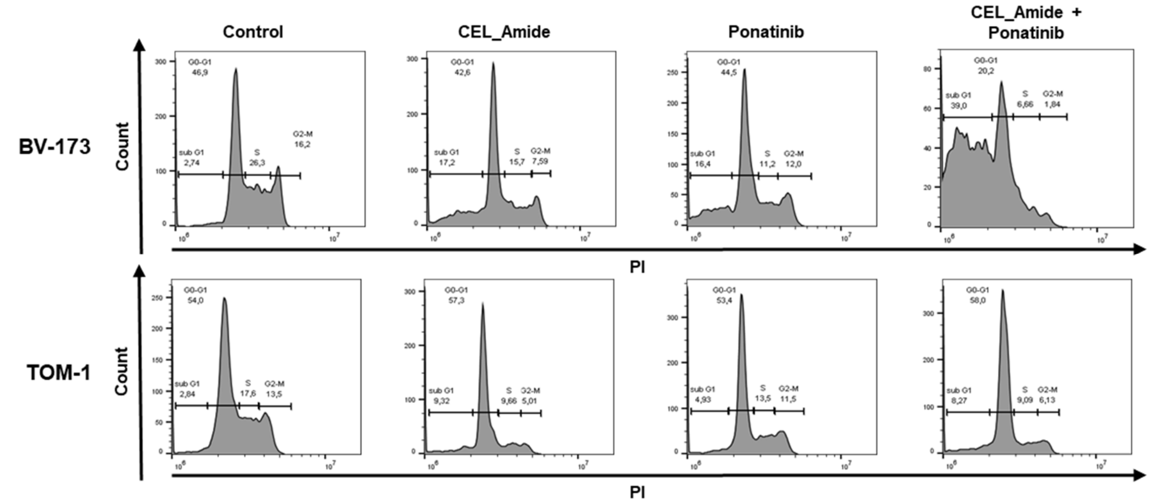

(B)

Figure S2: Effect of drug combinations with TKI on cell cycle in *BCR::ABL*+ ALL cell lines. Representative cell cycle flow cytometry of BV-173 or TOM-1 treated with 1500nM of CEL_Amide for 48h alone and in combination with 25nM of nilotinib (**A**) or 1nM of ponatinib (**B**). One representative experiment out of three is shown.

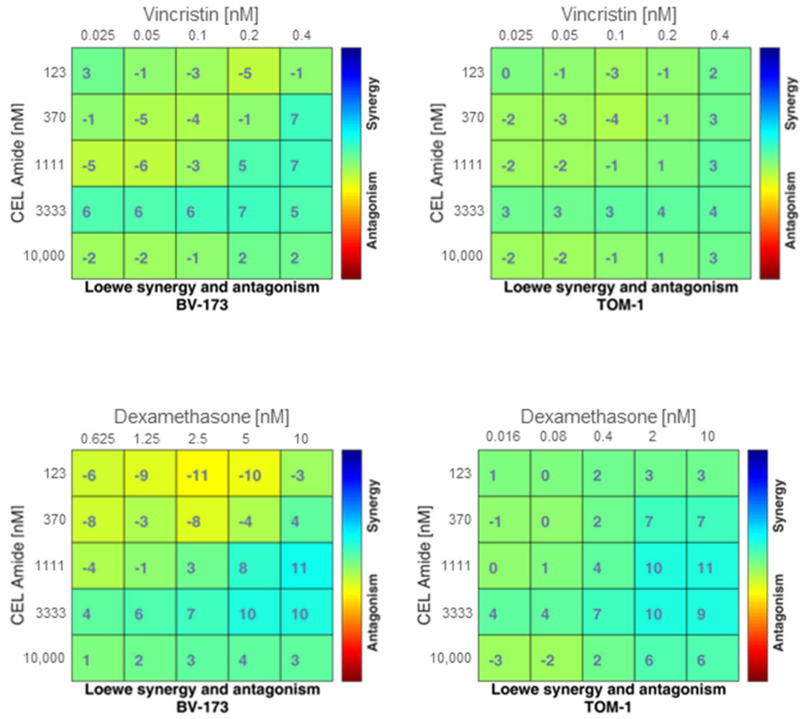

Figure S3: Drug combinations of LIMKi with vincristin and dexamethasone in *BCR::ABL* ALL cell lines. BV-173 and TOM-1 cell lines were exposed to increasing doses of vincristin (**A**) or dexamethasone (**B**) in combination with CEL_Amide. The dose-response matrix was made according to the Loewe model. Results are shown from duplicate of three independent experiments.

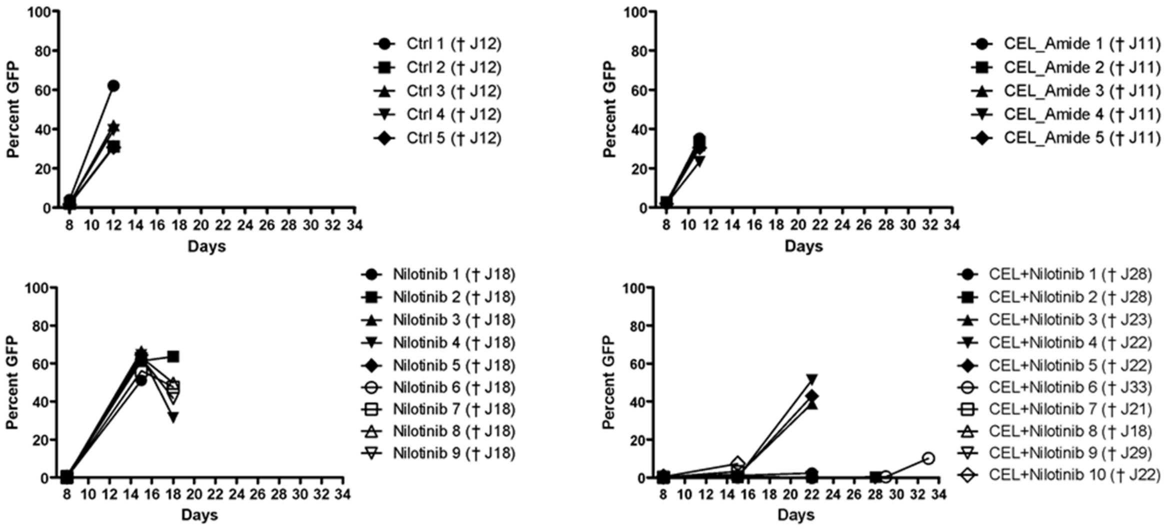

(A)

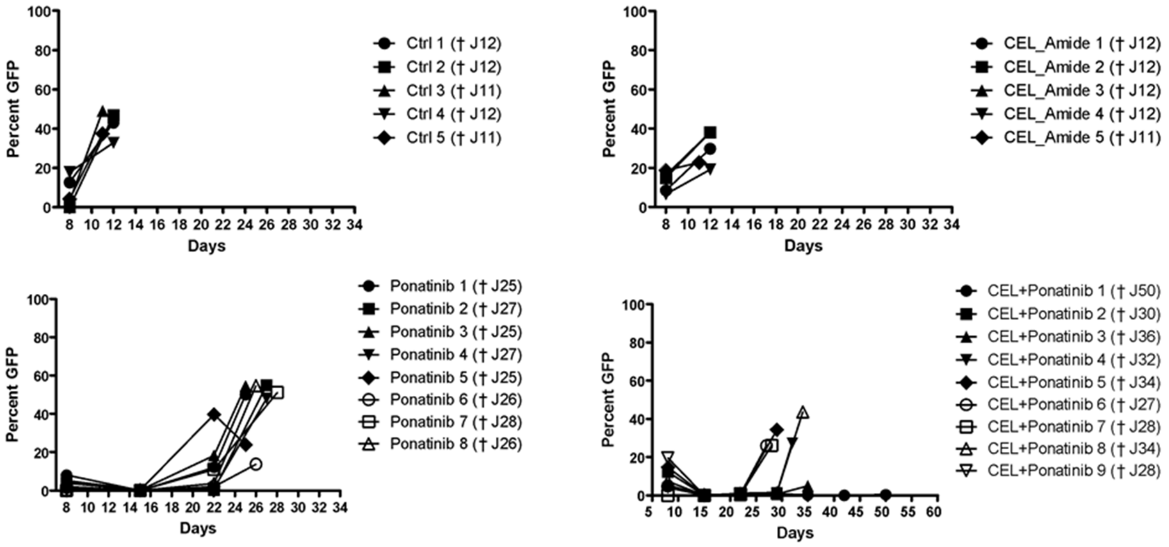

(B)

Figure S4: Detection of GFP positive *BCR::ABL*+ blast cells in C57BL/6J mice. Mice blood samples were collected at different time points. GFP+ cell percentage in control group, CEL_Amide group, nilotinib group and combination group are represented (**A**). GFP percentage in control group, CEL_Amide group, ponatinib group and combination group are represented (**B**).
